# Effect of High-Dose Vitamin D on Duration of Mechanical Ventilation in ICU Patients

**DOI:** 10.22037/ijpr.2019.1100647

**Published:** 2019

**Authors:** MirMohammad Miri, Mehran Kouchek, Alireza Rahat Dahmardeh, Mohammad Sistanizad

**Affiliations:** a *Department of Critical Care Medicine, Emam Hossein Medical and Educational Center, Shahid Beheshti University of Medical Sciences, Tehran, Iran.*; b *Department of Clinical Pharmacy, School of Pharmacy, Shahid Beheshti University of Medical Sciences, Tehran, Iran. *; c *Department of Pharmaceutical Care Unit, Emam Hossein Medical and Educational Center, Shahid Beheshti University of Medical Sciences, Tehran, Iran.*

**Keywords:** Vitamin D, Successful weaning, Mechanical ventilation, Intensive care

## Abstract

The relationship of vitamin D3 with the duration of mechanical ventilation and mortality is still unknown. Therefore, this study aimed to determine the effect of using high-dose vitamin D on the duration of mechanical ventilation among the patients admitted to the intensive care unit. The current double-blinded clinical trial was performed on 44 mechanically ventilated, adult patients. Using permuted block randomization, the patients were recruited in intervention and placebo arms. In the placebo group, four patients were excluded due to death before 72 h. The vitamin D level was measured in both groups on entrance and 7^th^ day of the study. The intervention and placebo groups received intramuscular injection of 300000 IU vitamin D and identical placebo, respectively. SOFA and CPIS score were evaluated daily for 7 days and on 14^th^ and 28^th^ days of the study. Also duration of mechanical ventilation and mortality rate were recorded. Fourteen males and 8 females were recruited in the intervention group, as well as 13 males and 5 females in the control group. There was no significant difference in baseline characteristics of the patients including gender and age. The mean duration of the mechanical ventilation was 17.63 ± 14 days in the intervention group versus 27.72 ± 22.48 days in the control group (*p* = 0.06). Mortality rate in control and intervention groups was 61.1% versus 36.3% (*p* = 0.00), respectively. Administration of high-dose vitamin D could reduce mortality in mechanically ventilated patients. Despite decrease of 10 days in duration of mechanical ventilation, the difference was not statistically significant. Larger studies are recommended.

## Introduction

Vitamin D is a fat-soluble vitamin, which is produced in the skin by UV radiation or supplied through diet. The association between vitamin D deficiency and increased risk of mortality has been indicated in non-critical patients (1-3). Since the first report of the vitamin D deficiency among the critically ill patients, in 2009, the high prevalence of its deficiency in the ICU patients, between 50 to 100 percent, has been shown in several studies (4-7).

In critically ill patients, the clinical outcome of vitamin D deficiency and role of its supplementation is not clear. One study reported that the vitamin D deficiency is directly related to the severity of the disease and the mortality rate, but it may not lead to prolonged duration of ICU stay, increased incidence of renal failure and sepsis (8-10). Contrary to these studies, many investigations reported that the vitamin D deficiency has a close relationship with prolonged hospitalization in the ICUs, re-admission, sepsis, and mortality (11-13). Some studies also exhibited that vitamin D deficiency could also increase the risk of respiratory failure (10, 14). 

Despite some studies done on the role of vitamin D in critically ill patients, there have been no controlled studies which evaluate the role of vitamin D in duration of mechanical ventilation. So, the main goal of the current study was to determine the effect of vitamin D supplementation on duration of mechanical ventilation among ICU patients.

## Experimental

The present single-centre double-blinded clinical trial was conducted on the patients admitted in the ICU of Imam Hussein Hospital after approval by the Deputy of Research of Shahid Beheshti University of Medical Sciences, Tehran, Iran. The sample size was estimated to be 44 according to previous studies and the equation for calculating the sample size (15). The patients were selected using convenient sampling method according to inclusion criteria and then divided into two groups, intervention and placebo, in 1:1 ratio by permuted block randomization. The participants in this study were recruited from adult (age between 18 and 65 years) mechanically ventilated patients. Exclusion criteria were refusal of the legal guardian of the patient to participate in the study, the patient′s death in less than 72 h after enrolling in the study, renal dysfunction (GFR < 30 mL/min), and the onset of dialysis during the study, vitamin D supplementation in the last 15 days, hypo/hyper-calcemia (adjusted calcium less than 8 mg/dL or above 10), liver failure (Child-Pough stage C), parathyroid dysfunction, CPIS (Clinical Pulmonary Infection Score) >6, INR > 1.5, platelet < 80,000 and hemodynamic disturbances (MAP less than 60 mmHg in three consecutive hours). Five mL venous blood was obtained on first day of mechanical ventilation, for determination of 25-OH vitamin D level. Then, the patients in intervention and placebo groups received intramuscular injection of 300,000 IU vitamin D and identical placebo, respectively. Both drug and identical placebo were prepared by Daroupakhsh Pharmaceutical Company, Tehran, Iran. Nurses and physicians were blinded to study groups.

**Table 1:** Baseline characteristics of the patients in control and intervention arms of the study

**Variables**	**Groups**		***p*** **-value**
	**Control (n = 18)**	**Intervention (n = 22)**	
Age (year)	56 ± 22.1	52 ± 22.1	0.55
Male gender (No.)	13	14	0.55
SOFA score baseline	6.6 ± 1.29	6.04 ± 0.95	0.85
CPIS score baseline	3.4 ± 1.6	3.13 ± 0.99	0.76
APACH II	19.77 ± 4.57	18.81 ± 4	0.49
VIT D (ng/dL) Patients with vitamin D deficiency (<30 ng/dL)	11.35 ± 18.23	8.43 ± 6.8	0.98

**Table 2:** Comparison of the mean duration of hospitalization, duration of mechanical ventilation and mortality of the patients in two groups

	**Group**		***p*** **-value**
	**Control (n = 18)**	**Intervention (n = 22)**	
Mechanical ventilation (days)	27.72 ± 22.48	17.63 ± 14	0.06
Length of ICU stay (days)	28.72 ± 23.58	19.5 ± 12.2	0.06
Mortality (%)	11 (61.1)	8 (36.3)	0.00

**Figure 1:**
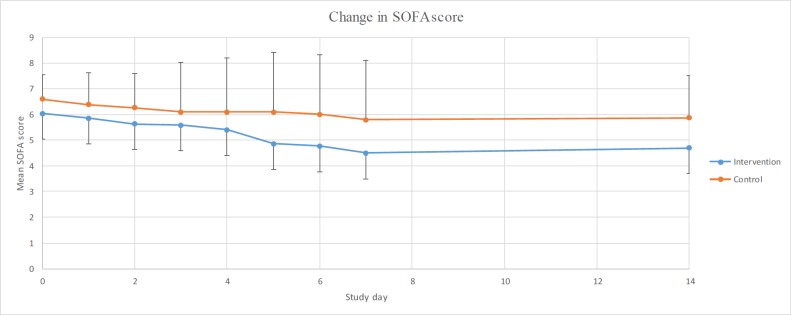
The changes in the mean SOFA scores in two groups over time

**Figure 2:**
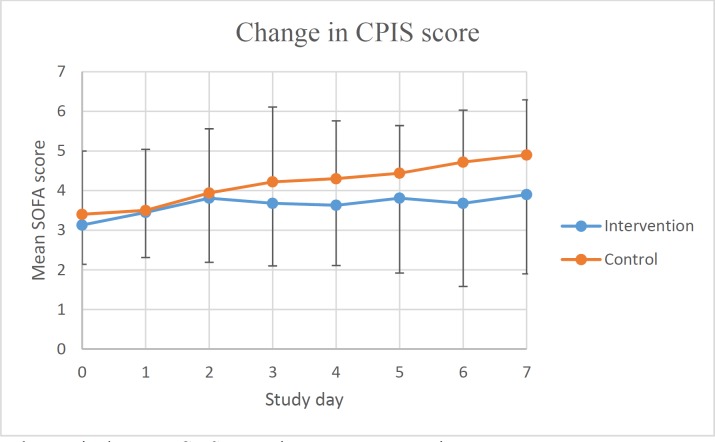
The changes in the mean CPIS scores in two groups over time

For all recruited patients, Acute Physiologic and Chronic Health Evaluation II (APACHE II) score was determined on the entrance to the study. Sequential Organ Failure Assessment (SOFA) score and Clinical Pulmonary Infection Score (CPIS) were assessed on daily basis until 7 days and then on 14^th^ and 28^th^ day of the study. Vitamin D level was measured on the 7^th^ day of the study. Moreover, the level of consciousness (using Glascow Coma Scale), duration of mechanical ventilation, length of ICU stay, and 28 day mortality were recorded in the pre-designed form for each patient. Data were analyzed by SPSS software version 24 using Chi-square, independent t-test, and repeated measures ANOVA and Wilcoxon test. Kolmogorov- Smirnov was used to check the normal distribution of the data. Mann-Whitney test was applied for non-parametric data. Otherwise, the independent t-test was used.

## Results

Totally, 44 patients were enrolled in the study, of which four were excluded from the study due to the death in less than 72 h who were in the control group. The remaining 40 patients included 27 males and 13 females (14 males and 8 females in the intervention group). There was no significant difference in gender between the two groups according to Chi-square test (*p* = 0.55). The mean age was 52 ± 22.1 and 56 ± 22.1 years in the intervention and control group, respectively (*p *= 0.55). In addition, there were no statistically significant differences among baseline characteristics of the patients in two arms of the study (Table 1).

Measurement of vitamin D levels on seventh day of the study showed an increase from 8.43 ± 6.80 to 10.48 ± 9.80 ng/dL in the intervention group and decrease from 11.35 ± 18.23 to 11.16 ± 18.22 ng/dL in the control group, but the difference between two groups did not reach to a significant level (*p* = 0.29). 

Duration of mechanical ventilation and length of hospital stay were 27.72 ± 22.48 *vs.* 17.63 ± 14.00 (*p* = 0.06) and 19.50 ± 12.20 *vs.* 28.72 ± 23.58 (*p* = 0.06) in control and intervention arms of the study, respectively. In addition, mortality rate in control and intervention group was 61.1% versus 36.3% (*p* = 0.00), respectively. Data are shown in Table 2.

The repeated measures ANOVA was used to evaluate the time effect that showed no significant difference in inter- and intra-group results in terms of SOFA scale in none of the situations (*p* = 0.13, *p *= 0.14). In addition, analysing daily changes in SOFA score compared to the baseline using repeated measures ANOVA showed no significant changes in the control group. However, this comparison revealed a significant decrease on the 5^th^, 6^th^, and 7^th^ day of the study in intervention group (*p*-values equal to 0.012, 0.006 and 0.002, respectively). Data are shown in Figure 1.

Changes in CPIS score over time were shown in Figure 2. Analysing daily changes in CPIS compared to the baseline using repeated measures ANOVA, showed that the mean score increased significantly in the control group (*p*-values were 0.47, 0.10, 0.00, 0.01, 0.00, 0.00 and 0.00, respectively), however, same analyses in the intervention group did not reach significant level. Despite different pattern of changes in two arms of the study, incidence of culture positive bacterial infections of respiratory tract did not show statistically significant difference (*p *= 0.74).

## Discussion

The results of the study showed that the mean days of mechanical ventilation decreased from 27 to 17 days in vitamin D group, but it did not reach statistical significance. Maybe the vitamin D deficiency could be one of the several culprits in the dependence on the ventilator and may prolong the time of weaning from the ventilator. In a study Quraishi *et al.*, measured plasma 25-hydroxyvitamin D levels in critically ill surgical patients on ICU admission and concluded that 25-hydroxyvitamin D levels were inversely associated with the duration of respiratory support (16). 

Another interesting finding of current study was effect of high dose vitamin D on all-cause mortality of the patients. Our results revealed that the number of survived patients was significantly higher in the intervention group than in the control. Therefore, high-dose vitamin D injections can increase the survival rate of the patients. 

The adequate vitamin D levels are necessary to regulate the function of the immune system, and its deficiency leads to an impairment of immune function. This could led to increased risk of infections, particularly ventilator-associated pneumonia, systemic inflammation, and multiple organ dysfunction syndromes which could increase mortality rate and length of ICU stay (17, 18). 

The results of vitamin D supplementation are controversial. In a study Aygencel *et al.* reported that the mortality rate was significantly higher in the vitamin D insufficient group compared to the vitamin D sufficient group but vitamin D deficiency in this study was not an independent risk factor for mortality (19). In another study, Putzu *et al.* revealed that vitamin D administration, in critically ill patients, could decrease the mortality without significant adverse events. In addition, Miroliaee, *et al., *showed that vitamin D supplementation, in patients with ventilator associated pneumonia, can significantly reduce the procalcitonin (20). Also, our results revealed that vitamin D supplementation could decrease mortality rate and ICU length of stay. In contrast to our results, Langlois *et al.*, in a systematic review concluded that Vitamin D administration does not improve clinical outcomes (7).

In our study despite one injection of 300,000 units vitamin D by IM rout, 25-OH vitamin D level did not increased significantly 7 days after administration. Amrein *et al.* showed that taking high oral doses in patients with vitamin D deficiency could improve its blood level within 2 days. In this study, 540000 units of oral medication were administered to the patients in the intervention group, which was more than the dose of our study. Unlike our results, they found an improved level of vitamin D above 25 ng/dL that did not match the results of this study. However, the effect of its improvement on the prognosis of patients was not evaluated (21). In another study Gorman *et al.* revealed that in subjects receiving a single high dose IM injection of vitamin D, serum 25-OH vitamin D levels increased at 3, 4, and 24 weeks post-injection, peaking at 4 weeks. This finding is in concordance with our results, which showed no increase in serum vitamin D level one week post-injection (22).

## Conclusion

This study showed that administration of high-dose vitamin D could be effective in reducing the duration of mechanical ventilation, the duration of hospitalization, and mortality rate in the ICU patients. Further studies with larger sample size and multi-centre design are required to support these conclusions.
